# Transitions into puberty and access to sexual and reproductive health information in two humanitarian settings: a cross-sectional survey of very young adolescents from Somalia and Myanmar

**DOI:** 10.1186/s13031-017-0127-8

**Published:** 2017-11-14

**Authors:** Anna E. Kågesten, Linnea Zimmerman, Courtland Robinson, Catherine Lee, Tenaw Bawoke, Shahd Osman, Jennifer Schlecht

**Affiliations:** 1 Department of Population, Family and Reproductive Health, Johns Hopkins Bloomberg School of Public Health, Baltimore, MD USA; 2 Department of International Health, Johns Hopkins Bloomberg School of Public Health, Baltimore, MD USA; 3 International Medical Corps, Addis Ababa, Ethiopia; 4 Women’s Refugee Commission, New York, NY USA; 5 Department of Public Health Sciences, Karolinska Institutet, Stockholm, Sweden

**Keywords:** Very young adolescents, Sexual and reproductive health, Refugees, Humanitarian settings, Somalia, Myanmar

## Abstract

**Background:**

Very young adolescents (VYA) in humanitarian settings are largely neglected in terms of sexual and reproductive health (SRH). This study describes the characteristics of VYA aged 10-14 years in two humanitarian settings, focusing on transitions into puberty and access to SRH information.

**Methods:**

Data were collected through a cross-sectional survey with Somali VYA residing in the Kobe refugee camp in Ethiopia (*N* = 406) and VYA from Myanmar residing in the Mae Sot and Phop Phra migrant communities in Thailand (*N* = 399). The average age was 12 years (about half were girls) in both communities. Participants were recruited using multi-stage cluster-based sampling with probability proportional to size in each site. Descriptive statistics were used to describe the sociodemographic, family, peer, and schooling characteristics and to explore transitions into puberty and access to SRH information.

**Results:**

Most VYA in both sites reported living with both parents; nine in ten reported feeling that their parents/guardians care about them, and over half said that their parents/guardians monitor how and with whom they spend their free time. High proportions in both sites were currently enrolled in school (91.4% Somali, 87.0% from Myanmar). Few VYA, particularly those aged 10-12, reported starting puberty, although one in four Somali indicated not knowing whether they did so. Most girls from Myanmar who had started menstruating reported access to menstrual hygiene supplies (water, sanitation, cloths/pads). No Somali girls reported access to all these supplies. While over half of respondents in both sites reported learning about body changes, less than 20% had learnt about pregnancy and the majority (87.4% Somali, 78.6% from Myanmar) indicated a need for more information about body changes. Parents/guardians were the most common source of SRH information in both sites, however VYA indicated that they would like more information from friends, siblings, teachers and health workers.

**Conclusions:**

This study highlights gaps in SRH information necessary for healthy transitions through puberty and supplies for menstrual hygiene in two humanitarian settings. VYA in these settings expressed closeness to their parents/guardians and the majority were in school. Introducing early SRH interventions that involve parents and educational centers may thus yield promising results, providing VYA with the necessary skills for understanding and dealing with their pubertal and sexual development.

**Electronic supplementary material:**

The online version of this article (10.1186/s13031-017-0127-8) contains supplementary material, which is available to authorized users.

## Background

The period of 10-14 years-of-age, generally defined as early adolescence, is one of the most critical stages of human development as marked by dramatic physical, social and cognitive changes [1, 2]. Yet, very young adolescents (VYA) are one of the most neglected populations in terms of research, particularly in the field of sexual and reproductive health (SRH) due to the combined sensitivity of the topic and the young age group [1–4]. Research and programmatic efforts in adolescent SRH have typically focused on the prevention of HIV and pregnancy among older adolescents (aged 15 years and above) while little is known about the specific SRH needs and concerns of VYA, including their evolving sexuality and fertility [1, 4–6]. Importantly, unsafe sexual behavior is one of the top causes of disability-adjusted life years among 10-14 year olds globally even though the majority has not yet engaged in sexual intercourse [7]. The burden of adverse SRH outcomes is especially high among young girls who, during the transition into adolescence, face increased risks of sexual coercion [8], early marriage, HIV and other sexually transmitted infections, and consequences due to early pregnancy [5, 7, 9]. Girls aged 14 years or younger account for almost one in three births (2 million out of 7 million) under the age of 18 every year and have double the risk for maternal mortality and morbidity than older peers [10]. In addition, sexually experienced VYA typically lack the information, skills and cognitive readiness needed to make informed decisions related to SRH including consensual sex and contraceptive use [3]. Evidence from sub-Saharan Africa shows that few parents discuss issues related to puberty and SRH with their 10-14 year olds, and VYA often lack access to sexuality education in schools [2]. Furthermore, in some contexts girls who have started puberty lack access to the supplies needed to manage their periods effectively [11] and according to a global systematic review many girls experience social stigma and shaming associated with menstruation [12].

While research on the SRH of VYA is gaining increased attention globally [13], little is known about the needs of refuge and migrant VYA in humanitarian settings, who are likely to be at particularly high risk of adverse SRH outcomes [14, 15]. Humanitarian emergencies, which generally include large-scale and long-term displacement, can disrupt family, peer, school, religious and other social support networks [16] – factors that are key influences on SRH and well-being during adolescence [1, 17]. The loss of support networks and exposure to new, often stressful, and insecure environments during emergencies and forced displacement can put VYA boys and girls at increased vulnerability to poverty, school-drop out, unsafe livelihoods, and violence including sexual exploitation and abuse; many girls may also face increased risks of early marriage [13, 16].

Historically, research among adolescents in humanitarian contexts is scant. In particular, research gaps persist with regard to studies on the needs and risks related to violence and SRH, given the sensitivity of these topics among VYA and the need to undertake such research with the highest ethical standards. Additionally, research on SRH in humanitarian settings remains challenged by the ongoing misperception that it is not as critical as other life-saving interventions (e.g. water, sanitation and nutrition) [18]. Available research also varies widely in quality, due to differences in data collection, analytic methods, and the limitations imposed by working in a humanitarian emergency [19]. As such, research on the SRH needs and factors affecting VYA in refugee and migrant settings remains nascent, despite our knowledge that SRH related issues are among the top causes of mortality and morbidity once this age group transitions into later adolescence.

A range of interconnected factors, whether in humanitarian settings or elsewhere, influences adolescent SRH. Blum et al. [1] describe a set of ecological-level factors at the macro, community, family, peer, and school levels that all shape the health and well-being in early adolescence. The Blum et al. framework can be used to better understand the transition to later adolescence and guide research and program development to meet the needs of VYA in humanitarian emergencies. However, before doing so, there must exist even a basic understanding of the context in which VYA in humanitarian settings grow up: their relationships to parents and peers; involvement with schooling or other activities; their experiences with pubertal transitions; and their knowledge of and preferred resources for information related to SRH. This research remains scarce, particularly in the peer-reviewed, scientific literature.

In response to this gap in research, the Women’s Refugee Commission and the Johns Hopkins Bloomberg School of Public Health, funded by the U.S. Centers for Disease Control and Prevention (CDC), and in partnership with three implementing agencies, International Medical Corps in Ethiopia, Save the Children in Lebanon, and Adolescent Reproductive Health Network in Thailand, explored the SRH needs and risks of refugee and migrant VYA through qualitative and quantitative research. This paper presents findings from a quantitative household survey with VYA implemented among displaced populations from Somalia (in Ethiopia) and Myanmar (in Thailand). We describe transitions into puberty (including pubertal status, reactions to pubertal changes, and menstrual hygiene access) among VYA in these two humanitarian settings, as well as whether and how they have access to SRH information related to body changes and pregnancy.

## Methods

Data for this study comes from the research project “Sexual and Reproductive Health Needs and Risks of VYA in Humanitarian Contexts” that took place in three different refugee or migrant settings in Ethiopia, Thailand and Lebanon. The project had three phases: 1) program and needs assessment, 2) qualitative research, and 3) quantitative research. The current study uses data from the quantitative research phase, which consisted of a cross-sectional survey about SRH risk and needs among VYA. The quantitative survey was carried out only in Ethiopia and Thailand; Lebanon was excluded due to security and capacity constraints faced by the local partner during the time of the quantitative research phase.

### Population

While the household survey was implemented in Thailand and Ethiopia, the populations of interest were VYA migrants from Myanmar and refugees from Somalia. Throughout the paper, we refer to the respondents in the Ethiopia refugee camp as “Somali VYA” and the respondents from the Thailand displaced and migrant communities as “VYA from Myanmar”.

In Thailand, the study population included migrant and displaced VYA from Myanmar residing in Mae Sot and Phop Phra districts – major crossing points on the Thailand-Myanmar border. Both communities are located in Tak Province, which hosts more than 120,000 migrants from Myanmar, a large proportion of whom are undocumented [20]. These two districts were chosen for the household survey because they represent prominent patterns of informal and undocumented migrant settlements and experiences that are important for understanding the needs of adolescents in this setting. Most migrants cross the border for domestic work, construction or fishing/ agriculture [21]. Adolescents in these areas may live with their families, or separately in boarding houses and shelters, given the transitory nature of work opportunities. Undocumented migrants on the Thailand-Myanmar border have a complicated and untrusting relationship with the Thailand authorities, and most have long-standing fears of visibility – which increases the risk for exploitation and abuse [20].

In Ethiopia, the study population included Somali VYA refugees residing in the Kobe refugee camp, which is part of the Dollo Ado refugee camp complex and has been hosting displaced Somali refugees since 2011. Somalia has been affected by a loss of government and internal conflicts for over two decades, leading to the displacement of over two million Somalis. Between 2011 and 2012, Somalia’s instability and armed conflict was compounded by drought and inflation [22]. The Kobe refugee camp covers 36 zones and hosts about 38,000 refugees displaced by crisis. According to the UN Refugee Agency (UNHCR) statistics, about 52% percent of the population in Kobe is female, and about 33% are younger than 17 years of age [23].

In both study sites, participants were selected through a multi-stage cluster sampling design. In Thailand, a total of 17 communities of migrants and displaced populations from Myanmar were identified using estimated population data from a variety of local, well-informed non-governmental and community-based organizations. From these 17 community population estimates, a total of 15 clusters were identified from Mae Sot and 5 from Phrop Phra with probability proportional to size. Each cluster was identified as comprising 20 households, selected in two steps: 1) randomly choosing one of a number of households known to include a family from Myanmar with at least one 10-14 year-old child, located close to a centralized starting point; and 2) using snowball sampling to identify the nearest next household and so forth until 20 households were identified for the cluster. If more than one cluster was identified in a particular community, two randomly selected “index” households were chosen at different start points in the community.

In Ethiopia, a total of 20 clusters (of 20 households each) were chosen from among 15 of the Kobe camp’s 36 existing zones using probability proportional to the size of the zone population data available from UNHCR. A centralized start was identified within each cluster (school, community center, temple/mosque/church) in collaboration with a community or zonal leader. Study teams then preceded in a random direction based on the spinning of a pen, and used a pre-established recruitment script (asking for households to identify other households with 10-14 year olds) to identify the 20 adolescents to be interviewed.

In both Thailand and Ethiopia, if a household had more than one 10-14 year old (which was true in a small number of cases), interviews were conducted with up to, but not more than, two randomly selected adolescents (representing both a boy and girl if possible).

### Procedure

After obtaining verbal parental consent and adolescent assent, respondents were interviewed face-to-face in the local language of each site. All respondents were informed that their participation was completely voluntary and that they could stop the interview or take a break at any time without consequences. Special care was taken during the training of interviewers to ensure correct translations of the original English survey questions into the local language. Interviews lasted about 45 min on average. The survey was developed based on the qualitative research conducted prior to the implementation of the quantitative phase, and contained seven domains: demographics, family, time use, school, peers, puberty and SRH information, and perceived safety and risk concerns. In Thailand, VYA were also asked about child labor and in Ethiopia the survey included a module about early marriage and personal/financial assets as these topics were identified as key in the qualitative research in each site.

Of note, during 40% of interviews in Ethiopia and 9% of interviews in Thailand the interviewers indicated that someone else in the household other than the adolescent insisted on being present at the time of the interview, though no detailed information was maintained as to whom that person was. Given the high numbers for which interview privacy was potentially compromised in Ethiopia, we conducted a sensitivity analysis by stratifying all analyses by whether someone else was present or not. The findings from the sensitivity analysis are presented in the results and a detailed overview is available in Additional file 1.

The study was approved by the Institutional Review Board (IRB) at the Johns Hopkins Bloomberg School of Public Health, by the Administration for Refugee and Returnee Affairs in Ethiopia, and by a community consultation team in Thailand.

### Measures

For the current study, we focused on variables related to sociodemographics, interpersonal relationships with parents and peers, school, pubertal transitions, and SRH information.

***Sociodemographic*** variables included *age* (10-12 vs. 13-14), *sex* (boy, girl), *place of birth* (camp/community, elsewhere in country, other country), *duration in each refugee or migrant camp* (years), and whether the respondent had a *regular place to stay* (yes, no).

***Family*** variables included whether the respondent’s *parents are alive* (both, mother only, father only, double orphan), *current living situation* (both parents, father or mother only, other or no adult caregiver), *number of children in the household*, and whether they *help care for those children* (yes, no). Two questions about *parental/guardian connectedness* measured the extent to which the respondents agreed or disagreed that their parents/guardians care about them, and that they can share personal matters (totally agree, agree, disagree, totally disagree). Two questions about *parental/guardian monitoring* assessed the extent to which the respondents believed that their parents/guardians know who their friends are and what they do with their free time (completely, some, very little, not at all). Items were analyzed continuously and further dichotomized to explore the proportion responding “totally agree” or “agree” versus “totally disagree” or “disagree” to each of the parental connectedness variables (parents/guardian care, parents/guardian listen), and the proportion responding “completely” or “to some extent” versus “not at all” or “very little” to each of the parental monitoring variables (parents/guardian monitor friends, parents/guardian monitor free time).

***Peer*** variables covered *number of close friends* (none, 1-2, 3 or more) and *opposite-sex close friends* (none vs. one or more). *Perceived peer norms about boy/girl relationships* were assessed by asking the respondent whether most of their friends think it is ok for boys and girls to: 1) “talk and spend time together”, 2) “spend time alone if they like each other”, and 3) “have physical contact if they like each other” (agree/disagree).

***School*** variables spanned *education status* (in school, out of school), *ever enrolled in school* (yes, no), *number of school days missed during the past week* (none, 1-2, 3-4 or 5 or more), and *perceived reasons that young people miss school* (e.g. lack of money, house work, parents do not approve).

***Pubertal transition*** experiences were assessed via a set of variables related to pubertal status, reaction to pubertal changes, and menstrual hygiene management (girls only). *Pubertal status* was conceptualized as starting menstruation among girls, and starting voice change or hair growth among boys (yes, no, do not know). *Reactions to pubertal changes* was assessed via three statements: “I like the fact that I am becoming a woman/man”, “I feel comfortable with the changes in my body”, and “My parents treat me differently now that I am becoming more like a woman/man” (dichotomized into totally agree/agree vs. disagree/totally disagree for each statement). Girls who reported starting menstruation were also asked about *access to menstrual hygiene supplies* such as water and soap, private washing facilities, and cloth pads (yes, no).

***SRH information*** included whether VYA reported that they had access to *information needed to understand body changes*, their demand for *more information about body changes* and whether they *learnt about pregnancy* (all with yes/no options). Those who reported starting puberty were also asked if they *learnt anything about puberty before these changes occurred* (yes/no). Respondents were asked to name their *most important information sources about body changes* and *from whom they would like to learn more about body changes* (mother, father, brother/sister, other relative, teacher, friend, religious leader, doctor/nurse, books/magazines, various media sources, other). In addition, two variables assessed *source of pregnancy information,* as well as whom they *usually seek health advice from* (mother, father, brother/sister, other relative, teacher, friends, religious leader, doctor/nurse, other).

### Statistical analysis

We conducted a descriptive analysis of the sociodemographic, family, peer and schooling characteristics for each of the two study sites, followed by a summary of the variables related to pubertal transitions and SRH information for girls and boys respectively. Categorical variables were reported as percentages, and means were calculated for continuous variables. All analyses used survey weights to account for the multi-stage cluster design; we also estimated 95% confidence intervals for each proportion and mean estimate (displayed in the tables). For the sensitivity analysis, we used Pearson’s chi-squared test to compare the proportion interviewed with someone else in the same room to those with no one else present for each variable. All analyses were conducted using Stata 12 (StataCorp, LP, College Station, TX, USA).

## Results

### Sociodemographics, family, peer and schooling characteristics

The final study sample comprised 406 Somali VYA and 399 VYA from Myanmar; the response rate was estimated at 95%. Table 1 summarizes the demographic, family, peer and school characteristics of the sample. The average age of respondents was 12.3 years among Somali VYA and 11.8 years among those from Myanmar; about half of respondents in both sites were girls (52.7% Somali, 50.4% from Myanmar). Almost all Somali respondents and about two thirds of those from Myanmar were born in another country. Most Somali respondents (95.8%) had resided in the Ethiopia refugee camp for less than five years, and in Thailand 57.9% of the respondents from Myanmar had lived in the displaced or migrant communities for five years or more. About one in three VYA from Myanmar reported not having a regular place to stay; the same was true for less than 2 % of Somali VYA.

**Table 1:** Demographic, family, peer and schooling characteristics of VYA from Somalia and Myanmar in two humanitarian settings

	Somali VYA^a^			VYA from Myanmar^a^		
	*N* = 406			*N* = 399		
	n	%	95% CI	n	%	95% CI
Demographics						
Sex						
Boy	192	47.3	(41.6, 53.0)	198	49.6	(43.1, 56.1)
Girl	214	52.7	(47.0,58.4)	201	50.4	(43.9, 56.8)
Age						
10-12	208	51.2	(42.7, 59.7)	262	65.7	(60.0, 70.9)
13-14	198	48.8	(40.3, 57.2)	137	34.3	(29.0, 39.6)
Mean age	12.3		(12.1, 12.5)	11.8		(11.6, 12.0)
Place of birth						
Camp/community	3	0.7	(0.0. 1.6)	109	27.3	(23.1, 31.6)
Elsewhere in country	–	–	–	22	5.5	(1.7, 9.3)
Other country	402	99.0	(98.0, 100.0)	264	66.2	(60.1, 72.2)
Do not know	1	0.3	(0.0, 7.6)	4	1.0	(0.0, 2.0)
Duration in camp/community						
1 year or less	7	1.7	(0.0, 3.6)	63	15.8	(10.7, 20.9)
2-4 years	382	94.1	(91.7, 96.5)	95	23.8	(18.9, 28.8)
5 years or more	1	0.3	(0.0, 0.8)	231	57.8	(52.1, 63.7)
Do not know	16	3.9	(1.8, 6.1)	10	2.5	(0.7, 4.3)
Regular place to stay	399	98.5	(97.1, 99.9)	270	67.7	(48.4, 87.2)
Interpersonal relationships						
Parents alive						
Both	335	82.5	(78.3, 86.7)	351	88.0	(84.7, 91.2)
Father only	18	4.4	(1.9, 7.0)	4	1.0	(0.0, 2.2)
Mother only	39	9.6	(6.3, 12.9)	31	7.8	(4.6, 10.9)
Double orphan	14	3.5	(0.7, 6.1)	13	3.3	(1.5, 5.0)
Currently living with						
Both parents	295	72.6	(67.0, 78.3)	249	62.4	(54.6, 70.1)
Father only	22	5.4	(2.4, 8.4)	14	3.5	(1.6, 5.4)
Mother only	63	15.5	(10.2, 20.8)	49	12.3	(7.5, 17.1)
Other adult caregiver	11	2.7	(0.5, 5.0)	74	18.6	(12.3, 24.8)
No adult caregiver	1	0.2	(0.0, 0.8)	2	0.5	(0.0, 1.2)
N/A	14	3.4	(0.0, 6.1)	11	2.8	(1.2, 4.4)
Nr of children in household	2.9		(2.6, 3.3)	2.0		(1.7, 2.3)
Help care for children in household	362	89.2	(82.5, 95.8)	235	58.9	(53.9, 63.9)
Parental/caregiver connectedness^b^						
Feel that parents/caregiver care	363	89.6	(82.5, 96.8)	364	91.2	(87.7, 94.7)
Feel that parents/caregiver listen	356	87.9	(82.4, 93.4)	286	71.7	(62.2, 81.2)
Parental/caregiver monitoring^c^						
Parents/caregiver know friends	205	50.7	(37.6, 63.8)	210	52.8	(44.4, 61.0)
Parents/caregiver monitor time	269	66.7	(56.4, 77.1)	214	53.6	(43.0, 64.3)
≥1 close friends	400	98.5	(97.4, 99.6)	346	86.7	(80.7, 92.7)
≥1 close opposite sex friend						
Boys (female friend)	79	41.1	(28.8, 53.5)	64	32.3	(19.0, 45.6)
Girls (male friend)	82	38.3	(26.2, 50.4)	45	22.4	(12.8, 31.9)
Perceived peer norms about boy/girl relationships						
Ok talk/spend time	163	40.1	(25.4, 54.9)	130	32.6	(20.9, 44.2)
Ok spend time alone	120	29.6	(15.5, 43.6)	93	23.3	(15.4, 31.1)
Ok physical contact	67	16.5	(6.5, 26.5)	50	12.5	(6.4, 18.7)
School						
Ever attended school	385	94.8	(91.7, 98.0)	385	96.5	(94.1, 98.9)
Currently enrolled in school	352	91.4	(87.7, 95.1)	335	87.0	(81.4, 92.6)
Days of school missed past week^d^						
None	201	57.1	(44.3, 68.9)	208	62.1	(53.0, 71.2)
1-2	106	30.1	(19.2, 41.0)	75	22.4	(16.0, 28.8)
3 or more	45	12.8	(5.5, 20.1)	52	15.5	(9.4, 21.6)
Perceived reasons that young people miss school^e^						
Lack of money	124	30.5	(17.8, 43.3)	284	71.2	(64.6, 77.7)
Distance to school	316	77.8	(69.6, 86.0)	125	31.3	(25.7. 37.9)
Safety concerns	45	11.1	(5.0, 17.1)	99	24.8	(16.9, 32.7)
Parent do not approve	140	34.5	(22.3, 46.7)	120	30.1	(23.7, 36.5)
House work	209	51.5	(39.3, 63.7)	162	40.6	(33.7, 47.6)
Marriage	108	26.6	(14.3, 38.8)	20	5.0	(2.5, 7.5)
Work	69	17.0	(8.8, 25.2)	183	45.9	(38.1. 53.6)
Health/disability	157	38.7	(24.7, 52.7)	147	36.8	(27.7, 45.7)
Other	16	3.9	(0.5, 7.4)	47	11.8	(7.3, 16.2)
Someone else present during the interview^f^	162	39.9	(22.7, 57.1)	26	6.5	(0.0. 13.2)

^a)^Somali VYA refer to the sample from the refugee camp in Ethiopia. VYA from Myanmar refer to the sample from the migrant communities in Thailand

^b)^Proportion agreeing with the statements “I feel that my parents/guardians care about me” (feel that parent/guardian care) and “I feel like I can share personal things with one of my parents/guardians and she or he will listen” (feel that parent/guardian listen), respectively

^c)^Proportion agreeing that their parents (to some extent or completely) know who their friends are (parents monitor friends), and what they do with their free time (parents monitor time), respectively

^d)^Days of school missed during past week were only asked of those currently enrolled

^e)^Respondents could select more than one reason for why they think people their age might miss school (total exceeds 100%)

^f)^In some cases it was not possible to obtain complete privacy during the interviews with the adolescents, as at least one other individual (unspecified) was present in the same room

Most respondents reported living with both parents (72.7% Somali vs. 62.4% from Myanmar), and some indicated living with a single parent (mother only: 15.5% Somali, 12.3% from Myanmar; father only: 5.4% Somali, 3.5% from Myanmar) or other adult caregiver (2.7% Somali, 18.6% from Myanmar). In both sites, less than 1 % reported living without an adult caregiver, and about 3 % were double orphans. Almost 90% of Somali VYA and two in three VYA from Myanmar reported that they help care for other children in the household. In both sites, nine in ten VYA reported feeling that their parents care about them (89.6% Somali, 90.4% from Myanmar), and high proportions that they feel comfortable sharing personal things with their parents (87.9% Somali, 71.7% from Myanmar). Two in three Somali VYA and half of those from Myanmar reported that their parents know how they spend their free time, and about half of respondents in both sites agreed that their parents know who their friends are.

Almost all Somali VYA (98.5%) and about nine in ten VYA from Myanmar (86.7%) reported having at least one close friend. Close friendships with the opposite sex were reported by about four in ten VYA from Somalia (38.3% of girls, 41.1% of boys) and by 22.4% of girls and 32.3% of boys from Myanmar. Similarly, about four in ten Somali VYA and a third of those from Myanmar indicated that most of their peers think it acceptable for boys and girls to talk and spend time together. Slightly lower proportions in both sites perceived that their peers approve of boys and girls spending time together alone without any adults around (29.6% Somali, 23.3% from Myanmar), and even fewer indicated peer approval of boys and girls having physical contact (16.5% Somali, 12.5% Myanmar).

School enrollment was high in both sites. The majority of respondents reported ever attending (94.8% Somali, 96.5% from Myanmar) and/or being currently enrolled (91.4% Somali, 87% from Myanmar) in school. About six in ten reported that they did not miss any school days during the past week; 12.8% of Somali and 15.5% of those from Myanmar indicated missing three or more days. When asked why young people their age might be unable to attend school, Somali VYA reported distance (77.8%), house work duties (51.5%), lack of money (30.5%), and marriage (26.6%) to be key barriers. The most commonly perceived barrier to school attendance among VYA from Myanmar was lack of money (71.2%), followed by requirements to work outside (45.9%) or inside (40.6%) the household. In both sites, one in four indicated health and disability concerns as key barriers to schooling, and one-third indicated that young people miss school because of lack of parental approval.

### Pubertal transitions

Table 2 shows pubertal development and reactions to pubertal changes among boys and girls in each of the two study sites.

**Table 2:** Pubertal transitions and access to SRH information

	Somali VYA^a^				VYA from Myanmar^a^			
	Boys		Girls		Boys		Girls	
	n	% (95% CI)	n	% (95% CI)	n	% (95% CI)	n	% (95% CI)
Pubertal status, by age								
10-12 years								
Did not start puberty	69	75.0 (58.3, 91.7)	82	70.7 (53.6, 87.8)	93	73.2 (64.0, 82.5)	118	87.4 (80.6, 94.2)
Started having periods (girls)			2	1.7 (0.0, 4.3)			16	11.9 (5.8, 17.9)
Started hair growth/voice change (boys)	1	1.1 (0.0, 3.2)			30	23.6 (13.6, 33.7)		
Do not know	22	23.9 (8.7, 39.2)	32	27.6 (10.7, 44.7)	4	3.2 (0.0, 7.5)	1	0.7 (0.0, 2.3)
13-14 years								
Did not start puberty	67	67 (48.5, 85.5)	64	65.3 (41.8, 88.8)	19	26.8 (13.2, 40.4)	22	33.3 (20.7, 46.0)
Started having periods (girls)			24	24.5 (4.9, 44.0)			44	66.7 (54.0, 79.3)
Started hair growth/voice change (boys)	15	15.0 (2.6, 27.4)			49	69.0 (55.4, 82.6)		
Do not know	18	18.0 (2.6, 33.4)	10	10.2 (0.0, 21.1)	3	4.2 (0.0, 8.9)	–	–
Pubertal change reactions^b^								
Like becoming a woman/man	14	87.5 (69.1, 100.0)	26	100.0 (−)	53	67.1 (54.1, 80.0)	36	60 (46.0, 74.0)
Feel comfortable with body changes	13	81.2 (62.4, 100.0)	25	96.2 (87.2, 100.0)	65	82.3 (70.3, 94.2)	39	65 (46.6, 83.4)
Treated differently by parents due to body changes	6	37.5 (16.7, 58.3)	21	80.8 (54.2, 100.0)	53	67.1 (53.6, 80.6)	36	60 (47.1, 72.9)
Menstrual hygiene access^b^								
Sufficient water and soap			16	61.5 (42.4, 80.6)			50	83.3 (72.1, 94.6)
Private washing facilities			5	19.2 (0.0, 38.7)			51	85 (76.1, 93.9)
Cloth/pads			5	19.2 (1.9, 36.6)			52	86.7 (76.7, 96.6)
All hygiene items			–	–			46	76.7 (65.9, 87.4)
SRH information								
Learnt about body changes before these occurred^b^	11	68.8 (49.9, 87.6)	25	95.2 (87.8, 100.0)	16	20.5 (12.4, 28.6)	19	31.7 (20.3, 43.1)
Access to information needed to understand body changes								
Disagree	50	26 (13.0, 39.1)	62	29.0 (13.5, 44.4)	106	53.5 (40.9, 66.2)	102	50.7 (41.3, 60.1)
Agree	136	70.8 (58.0, 83.7)	143	66.8 (51.3, 82.3)	70	35.4 (23.3, 47.4)	82	40.8 (31.4, 50.1)
N/A	6	3.1 (0.2, 6.0)	9	4.2 (0.0, 8.4)	22	11.1 (4.9, 17.3)	17	8.5 (4.6, 12.4)
Wish had access to more information about body changes								
Disagree	26	13.5 (5.4, 21.7)	22	10.3 (2.2, 18.4)	34	17.2 (9.1, 25.2)	34	16.9 (8.7, 25.2)
Agree	159	82.8 (73.8, 91.8)	187	87.4 (79.4, 95.4)	150	75.8 (65.0, 86.5)	158	78.6 (70.1, 87.1)
N/A	7	3.7 (0.0, 7.3)	5	2.3 (0.0, 4.7)	14	7.0 (2.1, 12.1)	9	4.5 (1.9, 7.0)
Learnt about pregnancy								
No	147	76.6 (64.2, 88.9)	173	80.8 (69.1, 92.5)	175	88.4 (80.6, 96.1)	173	86.1 (80.3, 91.8)
Yes	42	21.9 (9.6, 34.1)	39	18.2 (6.5, 30.0)	15	7.6 (3.2, 11.9)	28	13.9 (8.2, 19.7)
Do not know	3	1.5 (0.0, 4.0)	2	0.9 (0.0, 2.3)	8	4.0 (0.0, 11.5)	–	–

^a)^Somali VYA refer to the sample from the refugee camp in Ethiopia. VYA from Myanmar refer to the sample from the migrant communities in Thailand

^b)^Proportion agreeing with each statement among those who reported starting puberty (menstrual hygiene only asked of girls)

#### Somali VYA

In the Kobe refugee camp, the majority of respondents reported that they did not yet start puberty. Less than 2 % of girls aged 10-12 and 24.5% of girls aged 13-14 indicated that they got their period, and 1% of boys aged 10-12 and 15% of boys aged 13-14 reported voice change or hair growth. Of note, a about one in four Somali girls and boys aged 10-12 reported *not knowing* if they had reached puberty; the same was true for 10.2% of girls and 18.0% of boys aged 13-14 in this study site. Out of those few who reported starting puberty (*n* = 16 Somali boys, *n* = 26 Somali girls), all girls and 87.5% of boys reported that they like becoming women/men, and almost all girls (96.2%) and 80.1% of boys indicated that they felt comfortable with the changes in their bodies. The majority of Somali girls (80.8%) who started puberty indicated that their parents treat them differently due to their body changes; the same was true for less than half of Somali boys (37.5%). Girls who reported starting puberty were also asked a number of questions about their access to menstrual hygiene supplies. Less than two in three (61.5%) of Somali girls reported having sufficient water and soap and less than one in five (19.2%) reported access to private washing facilities (19.2%) and cloths or pads to use during menstruation. No Somali girls in this sample reported access to all three aspects of basic menstrual hygiene management.

#### VYA from Myanmar

In the Thailand migrant communities, 87.4% of girls and 73.2% of boys 10-12 years of age indicated that they had not started puberty. In contrast, 66.7% and 69.0% of 13-14 year old girls and boys reported having periods and hair growth/voice change, respectively. Among VYA from Myanmar who had begun these pubertal transitions, about two in three reported that they like growing up (becoming women/men) and indicated differential parental treatment as a result of their changing bodies (60.0% of girls, 67.1% of boys); 65.0% of girls and 82.3% of boys reported feeling comfortable with their body changes. Relatively high proportions of girls from Myanmar who started puberty reported access to menstrual hygiene supplies. The majority reported having sufficient water and soap (83.3%), access to private washing facilities (85.0%) and cloths or pads to use during menstruation (86.7%); overall, 76.7% reported access to all three resources to manage menstrual hygiene.

### Access to and sources of information about SRH

Table 2 also provides an overview of access to SRH information by site and gender, and Table 3 presents both important and preferred body change and pregnancy information sources for VYA.

**Table 3:** Information sources about SRH and general health

	Somali VYA^a^							
	Girls				Boys			
	Main puberty info source	Want more puberty info from	Pregnancy info source^b,c^	Seek health advice from^c^	Main puberty info source	Want more puberty info from	Pregnancy info source ^b,c^	Seek health advice from^c^
	(*n* = 214)	(*n* = 214)	(*n* = 39)	(*n* = 209)	(*n* = 192)	(*n* = 192)	(*n* = 42)	(*n* = 190)
	% (95% CI)	% (95% CI)	% (95% CI)	% (95% CI)	% (95% CI)	% (95% CI)	% (95% CI)	% (95% CI)
Mother	74.8 (64.4, 85.1)	19.2 (9.7, 28.7)	92.3 (76.5, 1.1)	83.1 (73.3, 93.0)	60.9 (48.5, 73.3)	10.9 (2.7, 19.2)	69.1 (47.3, 90.8)	79.7 (71.8, 87.6)
Father	9.4 (2.6, 16.1)	3.3 (1.1, 5.4)	33.3 (14.8, 51.8)	62.6 (51.8, 73.4)	17.2 (7.4, 26.9)	10.4 (3.6, 17.2)	52.4 (35.2, 69.5)	68.2 (59.8, 76.6)
Sibling	4.7 (1.5, 7.9)	19.6 (11.2, 28.0)	38.5 (22.0, 54.9)	59.3 (45.7, 72.9)	8.9 (5.4, 12.3)	26.6 (16.8, 36.3)	47.6 (32.5, 62.7)	54.2 (44.1, 64.2)
Other relative	0.5 (0.0, 1.5)	3.3 (0.8, 5.8)	12.8 (0.8, 24.8)	7.5 (0.9, 14.0)	1.6 (0.0, 3.9)	6.3 (0.2, 12.3)	11.9 (0.9, 22.9)	17.9 (10.3, 25.1)
Teacher	0.5 (0.0, 1.4)	7.5 (3.5, 11.4)	12.8 (0.0, 28.9)	8.8 (4.8, 13.0)	–	5.7 (1.6, 9.8)	31.0 (9.4, 52.4)	12.6 (6.6, 18.4)
Friend(s)	6.1 (1.2, 10.9)	32.2 (21.1, 43.3)	43.6 (16.3, 70.9)	39.2 (29.3, 49.2)	7.3 (0.3, 14.3)	25.0 (14.8, 35.2)	52.4 (27.7, 77.0)	34.7 (23.9, 44.8)
Religious leader	–	4.7 (0.0, 9.5)	2.6 (0.0, 7.8)	6.5 (2.1, 10.9)	0.5 (0.0, 1.6)	2.6 (0.4, 4.8)	4.8 (0.0, 12.2)	7.8 (3.1, 12.5)
Doctor/nurse	–	4.7 (0.0, 10.7)	–	18.2 (5.5, 30.9)	–	4.7 (0.0, 10.6)	2.4 (0.0, 7.3)	12.0 (2.8, 21.2)
Media^d^	0.9 (0.0, 2.9)	–			0.5 (0.0, 1.6)	0.5 (0.0, 1.6)		
Other	–	–	–	0.5 (0.0, 1.5)	0.5 (0.0, 1.6)	0.5 (0.0, 1.6)	–	2.1 (0.0, 4.6)
Do not know	–	1.4 (0.0, 3.0)	–		1.6 (0.0, 3.4)	3.1 (0.2, 6.0)	–	
Did not learn	3.3 (0.0, 9.1)	4.2 (0.0, 10.1)			1.0 (0.0, 3.2)	3.7 (0.0, 7.0)		
No one				1.9 (0.0, 5.7)				1.0 (0.0, 2.6)
	VYA from Myanmar^a^							
	Girls				Boys			
	Main puberty info source	Want more puberty info from	Pregnancy info source ^b,c^	Seek health advice from^c^	Main puberty info source	Want more puberty info from	Pregnancy info source^b,c^	Seek health advice from^c^
	(*n* = 201)	(*n* = 201)	(*n* = 28)	(*n* = 196)	(*n* = 198)	(*n* = 198)	(*n* = 14)	(*n* = 190)
Mother	47.3 (33.8, 60.7)	16.4 (9.9, 22.9)	57.1 (35.4, 78.9)	82.1 (13.2, 22.6)	23.2 (15.4, 31.1)	6.6 (1.6, 11.6)	40.0 (4.5, 75.5)	84.3 (78.3, 90.3)
Father	–	1.5 (0.0, 3.2)	3.6 (0.0, 10.6)	36.3 (26.8, 45.8)	10.1 (4.0, 16.2)	2.0 (0.0, 4.5)	33.3 (10.2, 56.7)	58.6 (47.3, 69.9)
Sibling	2.0 (0.0, 3.9)	5.0 (1.9, 8.1)	21.4 (6.1, 36.8)	31.8 (25.0, 38.7)	3.0 (0.5, 5.6)	4.0 (1.2, 6.9)	6.7 (0.0, 20.5)	22.1 (15.6, 26.9)
Other relative	3.5 (2.1, 5.7)	3.0 (0.7, 5.2)	14.3 (0.0, 30.2)	15.9 (10.6, 21.3)	3.0 (0.0, 6.1)	3.0 (0.4, 5.7)	20.0 (0.0, 42.2)	15.2 (8.6, 21.7)
Teacher	12.4 (6.1, 18.8)	31.8 (24.3, 39.3)	64.3 (50.1, 78.0)	45.3 (36.8, 53.8)	15.1 (8.2, 22.0)	34.9 (25.2, 44.5)	66.7 (43.5, 89.8)	43.9 (37.1, 50.7)
Friend(s)	5.0 (2.2, 7.7)	7.5 (4.7, 10.2)	28.6 (13.3, 43.8)	33.3 (25.0, 41.7)	5.6 (1.0, 10.1)	6.6 (2.4, 10.7)	20.0 (0.0, 45.8)	19.7 (12.4, 26.9)
Religious leader	–	1.5 (0.0, 3.3)	–	2.5 (0.0, 5.0)	1.0 (0.0, 2.5)	2.5 (0.0, 5.0)	–	0.5 (0.0, 1.6)
Doctor/nurse	0.5 (0.0, 1.5)	14.9 (10.9, 18.9)	32.1 (10.4, 53.8)	28.9 (18.0, 39.7)	6.1 (2.1, 10.0)	16.7 (9.7, 23.6)	46.7 (21.8, 71.5)	27.8 (16.3, 39.2)
Media^d^	3.0 (0.0, 6.5)	7.5 (0.3, 12.1)			6.6 (0.3, 12.8)	10.1 (3.8, 16.3)		
Other	0.5 (0.0, 1.5)	0.5 (0.0, 1.5)	21.4 (8.5, 34.3)	2.9 (0.4, 5.6)	–	0.5 (0.0, 1.6)	13.3 (0.0, 31.4)	4.0 (0.7, 7.4)
Do not know	1.0 (0.0, 2.4)	7.0 (2.5, 11.4)	–	–	1.5 (0.0, 3.3)	4.0 (0.5, 7.6)	–	–
Did not learn	24.9 (8.5, 41.2)	3.5 (0.6, 6.3)			24.8 (6.3, 42.1)	9.1 (3.8, 14.3)		
No one				10.9 (4.3, 17.6)				11.6 (3.9, 19.3)

^a)^Somali VYA refer to the sample from the refugee camp in Ethiopia. VYA from Myanmar refer to the sample from the migrant communities in Thailand

^b)^Asked of those who reported learning about pregnancy

^c)^Respondents could select more than one option (total exceeds 100%)

^d)^Media includes books/magazines, films/video and Internet

#### Somali VYA

Almost all Somali girls and about two in three boys who started puberty indicated having learnt about body changes before these occurred (95.2% girls, 68.8% boys), and two thirds agreed that they have access to the information needed to understand body changes (66.8% girls, 70.8% boys). However, high proportions of both girls and boys wished they had access to more information about body changes (87.4% girls, 82.2% boys) and less than one in four reported learning about pregnancy (18.2% girls, 21.9% boys) (Table 2).

In the Kobe refugee camp, mothers were the most commonly reported source of information for pubertal body change among girls (74.8%) followed by her father (9.4%) and her friends (6.1%). However, Somali girls reported that they would like to learn more about body changes from their friends (32.2%) in addition to their mother (19.2%) and siblings (19.6%). Most Somali boys reported that they learnt about puberty from their mothers (60.9%) followed by father (17.2%) and siblings (8.9%). Boys in this site reported that they would primarily like more information from siblings (26.6%) or friends (25%) followed by mother (10.9%) or father (10.4%). Somali VYA who reported learning about pregnancy indicated that they did so from their mother, father (boys only), friends and brothers/sisters (girls only). When asked whom they would turn to for advice about their health, Somali respondents largely listed family members (mother, father, siblings) or friends.

#### VYA from Myanmar

In the migrant communities in Thailand, about one in three girls and one in five boys who started puberty said that they learnt about body changes before these occurred, and less than half (40.8% girls, 35.4% boys) agreed that they can access the information needed to understand the changes in their bodies; almost eight in ten (78.6% girls, 75.8% boys) indicated a need for more information on this topic. Very low proportions of VYA from this study site reported learning about pregnancy (13.9% girls, 7.6% boys).

VYA from Myanmar further reported that their three most important sources for learning about puberty were: mother (47.3% girls, 23.2% boys), teachers (12.4% girls, 15.1% boys) and father among boys (10.1%). About one in four respondents stated that they did not learn about puberty and therefore did not report an information source. Both girls and boys reported that they would like to learn more about body changes from teachers (31.8% girls, 34.9% boys), doctors/nurses (14.9% girls, 16.7% boys) and media (boys only, 10.1%). Girls (but not boys) also reported their mother as a key preferred source (16.4%). Those who reported learning about pregnancy said that they received this information from their teachers (64.3% girls, 71.4% boys), doctors/nurses (32.1% girls, 50.0% boys), mother (57.1% girls, 42.9% boys), and father (boys only, 35.7%). Respondents in these migrant communities also indicated that they would first and foremost turn to their mother, fathers and teachers for health advice.

### Sensitivity analysis of interview privacy with Somali VYA

Results from the sensitivity analysis indicated that Somali VYA for whom someone else was present during the interview (40%) differed in their responses on some of the background variables when compared to those interviewed in complete privacy (Additional file 1, Table 1). Those with someone else present were somewhat more likely to live with both parents (77.2% vs. 69.7%, *p* = 0.011) and to report that their parents/guardian care about them (96.3% vs. 85.2%, *p* < 0.001), but less likely to say that their parents monitor their time (55.9% vs. 73.4%, *p* = 0.028). They were also borderline more likely to report peer norms supporting boys and girls having physical contact (26.5% vs. 9.8%, *p* < 0.05). No significant differences were found for the other background variables (e.g., sex, age, place of birth, duration in camp, having a regular place to stay, number of close friends, schooling).

With regards to pubertal transitions and access to SRH information, there were generally no significant differences between those interviewed with or without someone else present (Additional file 1, Table 2). The conference intervals were wide due to the small cell numbers, and the results should overall be interpreted with caution. There was some indication that more girls aged 13-14 years who were interviewed in the presence of someone else reported that they started puberty (*n* = 16 vs. *n* = 8), and higher numbers of those that started puberty reported that their parents treat them differently (*n* = 17 vs. *n* = 4); these associations were however only borderline significant (*p* < 0.1). Girls interviewed with someone else were also more likely to report having learnt about pregnancy (31.8% vs. 8.7%, *p* = 0.016), while the same was only marginally significant among boys (33.8% vs. 14.4%, *p* = 0.094). The top information sources about SRH were generally similar when comparing those interviewed in the presence of someone else to those who were not (Additional file 1, Table 3a-b). There was however some indication that higher proportions of girls and boys interviewed with someone else present preferred to get more information about puberty from doctors or nurses, and lower proportions of boys wanted more information from their fathers.

## Discussion

Adolescence is a time marked by rapid physical, emotional, cognitive and social change. For adolescents in humanitarian crises, these changes often coincide with family separation, disruption of school, the breakdown of family and other social structures that, in more stable times, are protective [14]. This study provides information on the interpersonal relationships and schooling of VYA growing up in two humanitarian settings, with a focus on their transitions through puberty and information about SRH, in order to better understand experiences that may impact their long term health and well-being. The study took place in two different humanitarian settings that varied with regard to length of displacement and type of settlement. In Ethiopia, the Somali VYA interviewed were primarily recent refugees living in the densely populated camp for less than five years, while in Thailand the adolescents interviewed from Myanmar lived in migrant communities that are long established; nearly half of respondents were born in Thailand and the majority have resided there for at least five years.

Our findings provide insight into the mechanisms of early sexual health education, and the extent to which VYA are exposed to information about their bodies and SRH in these two humanitarian settings. While access to SRH information appeared to be relatively low among migrant VYA from Myanmar, Somali VYA in the Kobe camp appeared to have relatively high exposure to such information as well as positive reactions to pubertal changes and the transition to adulthood (this was true among both boys and girls). Additionally, this information appeared to be routinely conveyed to Somali VYA before their transition through puberty, and often so by their fathers or mothers. Nevertheless, about one in four Somali boys and girls indicated that they did not know if they had actually entered puberty. This could be explained by late onset of menarche, embarrassment or shyness, or that information that is being shared is either incomplete or inaccurate. In addition, one third of Somali and half of respondents from Myanmar indicated that they did not have access to information about body changes, less than 20% reported learning about pregnancy, and the majority in both sites expressed a need for more advice on puberty. This signals the need for educational efforts to start early, occur often, and continue through the course of adolescence.

Mothers and fathers appeared to be a key information source for learning about puberty in both sites. Few respondents in this study were double orphans, and while some had lost their mother or father, most reported living with at least one of their parents. The importance of family structure for adolescent SRH was recently demonstrated by a study in rural Sierra Leone, which found that adolescents aged 13-19 years who lived with their mother or both parents had lower odds of recent sexual activity compared to those who did not [24]. In the current study both boys and girls – particularly those from Somalia - also reported high levels of parental connectedness and monitoring, signaling that parents continue to play a large role in VYA’s lives at this stage. These findings are important given the essential role of parents in promoting health, well-being and resilience among adolescent refugees through parent-child support and communication [25, 26]. Parental connectedness and monitoring have also been found to serve as key protective factors for sexual risk taking including early and unsafe sexual behaviors [17]. Programs that involve and support parents to communicate with their adolescent children, and provide in-depth information about the physical and emotional changes that occur in the early adolescent period, can be beneficial [26]. For example, although not implemented in a humanitarian setting, a program conducted in Uganda to improve SRH knowledge in primary schools used a holistic approach that involved parents among other stakeholders in adolescent health, and showed positive results among its evaluation with a marked increase in the knowledge of these adolescents about prevention of STIs and pregnancy [27]. Since parents were clearly a source of SRH information to the VYA in the current study, it is important for programmers to consider what role parents can play in SRH interventions that take place in humanitarian settings.

Some VYA reported that while they may have learned about SRH from their families (primarily mother or father), they would like to receive more information from other sources. The majority of VYA from Myanmar preferred receiving information from a source outside of the family, such as teachers whom VYA appeared to trust and from whom they sought for information. Little is known, however, about whether teachers in migrant schools and schools in other humanitarian settings feel equipped to provide this information, and whether teachers receive training in comprehensive sexuality education given that the latter is key for high-quality sexuality education [28]. Nevertheless, education centers could be central for conveying information about SRH to adolescents, especially when enrollment is high, and parents are involved in the development of such programs and thus endorse or support them (though this may depend on whether populations are settled in camps and concentrated settlements or more broadly scattered among host communities). In the qualitative phase of research preceding the current study, schools and staying in school were identified as extremely important to both VYA and their parents (see Ortiz et al. [29] and Lee et al. [30] in this Journal supplement); findings that are supported by the high school enrollment for both boys and girls across both study sites in the current study. Unfortunately school-based programs will miss the most vulnerable, out-of-school adolescents, so it would be important to have complementary methods for reaching out-of-school VYA if this were an approach taken.

Increasingly, humanitarian actors are acknowledging the importance of addressing menstrual hygiene management (MHM) within emergencies, in order to promote school attendance, reduce isolation and improve dignity of girls and women [31]. MHM is particularly important for adolescents, as they are at highest risk of missing school or dropping out because of poor MHM options [11]. Despite the inclusion of MHM across many sectors within the Sphere 2011 guidelines [32], it is widely overlooked. Our study findings confirmed that lack of MHM resources is particularly true among Somali refugees. In the Ethiopia refugee camp studied, very high proportions of girls reported lacking access to the most basic aspects of MHM: access to soap and water, a private wash facility, and sanitary materials such as cloths or pads. None of the girls in this setting had access to all three in contrasts to the Thailand migrant communities, where more than 70% of girls reported access to all three elements. The relatively high access in Thailand to menstrual hygiene supplies may be impacted by the longevity of these migrant communities, where the population can benefit from locally established water and sanitation infrastructures. New guidelines on the response to MHM-related needs in humanitarian settings are currently in development by a consortium led by the International Rescue Committee [33]. These guidelines should provide meaningful direction for how to respond to MHM-related needs including water, sanitation and hygiene, education and reproductive health, as well as how to dismantle local and cultural taboos associated with menstruation and its management. In addition, such guidelines can serve as a platform for advocacy and training around MHM in humanitarian settings. As noted by Sommer [31], addressing the issue of menstrual hygiene management in low-resource areas is however complex, and requires inter-sectorial collaboration and clarification of roles across water, health, education and urban planning.

In designing humanitarian programming for this population, it is critical to understand their needs, as well as barriers faced in attending structured interventions. The fact that one-third of VYA from Myanmar indicated that they do not have a regular place to stay, and that 13% were not currently enrolled in school, speaks to a level of instability that persists years after displacement for this particular population. Outreach and consistent engagement may be particularly challenging for this group. It is also important to note the high percentage of VYA who reported caretaking responsibilities for younger siblings, especially in the Somali community. This competing responsibility could significantly affect the ability to engage VYA in programming.

Given these initial findings it is important that we continue to invest in research and programming for VYA in humanitarian settings. Indeed, while descriptive, data from the current study about parental and peer relationships, as well as sources of SRH information, challenge assumptions about traditional gendered societies. Findings from the Somali refugee camp in Ethiopia indicated great potential for engagement of mothers and fathers in education around sensitive topics. Additionally, that fact that over a third of Somali boys and girls reported mixed-gender peer relationships contrast the qualitative findings presented as part of this journal supplement. In the study by Ortiz et al. [29] gender roles in the Kobe camp were described as highly unequal, with relationships between boys and girls becoming more restrictive with the onset of puberty and only considered acceptable within the context of marriage. However, there were also indications from adult men and women that such gender norms are changing towards more equitable as a result of displacement. More research is thus needed to better understand whether and how perceptions of gender norms change following displacement and over the course of adolescence.

Overall, our findings indicate that Somali VYA and those from Myanmar are growing up in unique contexts and that programs designed to ensure healthy transitions into adolescence needs to be tailored according to the specific needs and preferences of these populations. These findings parallel recommendations from the United Nations Population Fund (UNFPA) toolkit for adolescent reproductive health in emergencies, which states that SRH programs “must take innovative approaches to make services acceptable, accessible and appropriate for adolescents, taking cultural sensitivity and diversity into consideration” [14]. However, more research is needed for the humanitarian community to broadly understand whether the site-specific findings in the current study are cultural, or to what extent they are the result of specific impacts of conflict: length of displacement, family separation, or camp versus non-camp communities.

### Strengths and limitations

The literature about factors affecting VYA sexual and reproductive health is scant. While the current study adds to the body of knowledge regarding this population, the study has several limitations. First, we were unable to compare our estimates to non-humanitarian settings and we thus do not know whether the findings are unique to VYA growing up in humanitarian settings or if similar results would have been found had the study been conducted in other resource-poor settings. Evidence indicates that adolescents growing up in humanitarian settings are at particularly high risk of adverse SRH settings [14, 15]; however given that most VYA in the current study reported many of the factors that are protective for SRH (e.g. parental connectedness, high school enrollment) it is possible that SRH disparities between humanitarian and non-humanitarian settings becomes apparent only later in adolescence. Secondly, because of the vulnerability of the respondents (given their very young age and the humanitarian context), we were unable to include questions about VYA personal attitudes and behaviors related to romantic and sexual experiences. Rather, in an attempt to reduce social desirability bias, questions were expressed in general terms or phrased in reference to what most of their friends think about the certain statements rather than asking the respondent about their own opinion. Given our conservative approach, it is possible that we missed valuable information regarding the depth of and need for SRH knowledge among VYA in these humanitarian settings.

Third, while attempts were made to ensure complete privacy, this was not possible for all respondents, especially in the Kobe refugee camp where for 40% of interviews, someone else was present in the same room. In the Kobe camp, both emergency tents and transitional shelters are designed as single room dwellings, with refugees themselves hanging curtains or dividers as available to divide the small space [34]; in this context it may not have been feasible to obtain full privacy during the interview. While we do not have information on *who* was present or whether they overheard the interview, the sensitivity analysis indicated that those who lacked privacy were more likely to live with both parents as well to report feeling that their parents/guardian care about them, and none were orphans. This may indicate that the likelihood that a parent or other guardian wanted to remain close at hand while the interview was carried out, or worried about leaving their child alone with the interviewer, was higher for these respondents. However, while it would be expected that sensitive issues such as SRH information would be underreported if someone else was nearby [35], our results indicated the opposite: VYA for whom someone else was present were more likely to report permissive peer norms about boy-girl relationships, to have learnt about pregnancy, and borderline more likely to indicate that their parents treat them differently due to pubertal body changes. A potential explanation for this could be that a parent or other adult helped explain the questions or provided additional information (for example regarding puberty). Indeed, it is possible that the physical proximity of a trusted adult was a source of reassurance rather than distress for the respondent [36, 37]. While the lack of privacy may have biased some of the responses, social desirability bias rending incorrect responses to questions on sensitive behaviors is an *overall* issue in surveys with adolescents. Because both underreporting and over-reporting can occur we also cannot determine whether the rates among those interviewed with someone else present are more or less “true” than those interviewed in complete privacy [35]. Further research is needed to determine the effect of having a parent or someone else present during interviews with VYA in humanitarian settings.

Finally, while some of the confidence intervals were wide due to the cluster-based design, this was a descriptive study and thus our sample was not powered to test specific hypotheses. In light of its limitations and descriptive nature, our study included over 800 refugee and migrant VYA and to our knowledge it is one of few studies that focused on the SRH needs of this population in humanitarian settings. Our findings thus provide an important baseline for the design of future research studies as well as interventions.

## Conclusions

VYA in humanitarian settings remain a much-neglected group in terms of SRH research. The current study showed that high proportions of Somali VYA living in an Ethiopian refugee camp and VYA from Myanmar living in displaced and migrant communities in Thailand were not receiving the information they need to understand transitions into adolescence, and menstrual hygiene management was a pressing concern among Somali VYA. SRH interventions that empower VYA with information and skills related to their developing bodies, sexualities and relationships offer opportunity to impact later SRH outcomes. In order to reach VYA in humanitarian contexts before they engage in sexual relationships, interventions need to move beyond the traditional health care system and tap into VYA existing learning networks. Broader intervention approaches may involve the strengthening of protective factors, such as parental connectedness, rather than a sole focus on risk factors that affect SRH. VYA in these humanitarian settings expressed closeness to their parents and most were enrolled in school; introducing interventions at those levels may yield promising results, helping them in their sexual and relationship decision-making. The results from the current study can help direct policy and program developers towards the best modalities for enabling VYA to access developmentally appropriate information and support, which in turn can help promote their sexual health and well-being throughout adolescence.

## Additional file

Additional file 1: Sensitivity analysis of interview privacy. (DOCX 166 kb)
