# Supplementary material for: Transitions into puberty and access to sexual and reproductive health information in two humanitarian settings: a cross-sectional survey of very young adolescents from Somalia and Myanmar
Source: Confl Health. 2017 Nov 14;11(Suppl 1):24. doi: 10.1186/s13031-017-0127-8 (PMC5688464; doi:10.1186/s13031-017-0127-8)
Supplement: Additional file 1: — Sensitivity analysis of interview privacy. (DOCX 166 kb) [file 13031_2017_127_MOESM1_ESM.docx]

**Additional file 1: Sensitivity analysis of interview privacy**

**Additional file, Table 1.** Demographic, family, peer and schooling characteristics of VYAs from Somalia: Sensitivity analysis by whether someone else was present during the interview (N=406)

|  | **No one else present** | | **Someone else present** | |  |
| --- | --- | --- | --- | --- | --- |
|  | N=244 | | N=162 | |  |
|  | n | % (95% CI) | n | % (95% CI) | *p** |
| **Demographics** |  |  |  |  |  |
| Sex |  |  |  |  |  |
| Boy | 118 | 48.4 (40.5, 56.2) | 74 | 45.7 (38.0, 53.3) | 0.616 |
| Girl | 126 | 51.6 (43.7, 59.5) | 88 | 54.3 (46.6, 62.0) |  |
| Age |  |  |  |  |  |
| 10-12 | 114 | 46.7 (36.7, 56.7) | 94 | 58.0 (46.5, 69.5) | 0.112 |
| 13-14 | 130 | 53.3 (43.2, 63.3) | 68 | 42.0 (30.5, 53.5) |  |
| Place of birth |  |  |  |  |  |
| Camp/community | 3 | 1.2 (0.0, 2.6) | – | – | 0.150 |
| Other country | 241 | 98.8 (97.3, 100.0) | 161 | 99.4 (98.0, 100.0) |  |
| Do not know | – | – | 1 | 0.6 (0.0, 1.7) |  |
| Duration in camp/community |  |  |  |  |  |
| 1 year or less | 2 | 0.8 (0.0, 2.5) | 5 | 3.1 (0.5, 6.1) | 0.213 |
| 2-4 years | 229 | 93.8 (90.7, 97.0) | 153 | 94.4 (91.8, 97.8) |  |
| 5 years or more | 1 | 0.4 (0.0, 1.2) | – | – |  |
| Do not know | 12 | 4.9 (1.9, 7.9) | 4 | 2.5 (0.0, 5.0) |  |
| Regular place to stay | 238 | 97.9 (95.9, 100.0) | 161 | 99.4 (98.1, 100.0) | 0.232 |
| **Interpersonal relationships** |  |  |  |  |  |
| Parents alive |  |  |  |  |  |
| Both | 197 | 80.7 (76.0, 85.5) | 138 | 85.2 (78.1, 92.3) | 0.142 |
| Father only | 10 | 4.1 (1.3, 6.9) | 8 | 4.9 (1.0, 8.9) |  |
| Mother only | 23 | 9.4 (5.8, 13.1) | 16 | 9.9 (2.9, 1.7) |  |
| Double orphan | 14 | 5.7 (1.4, 10.1) | – | – |  |
| Currently living with |  |  |  |  |  |
| Both parents | 170 | 69.7 (62.9, 76.4) | 125 | 77.2 (69.9, 84.8) | **0.011** |
| Father only | 14 | 5.7 (2.0, 9.5) | 8 | 4.9 (1.1, 8.7) |  |
| Mother only | 39 | 16.0 (9.9, 22.1) | 24 | 14.8 (7.2, 22.5) |  |
| Other adult caregiver | 7 | 2.9 (0.0, 5.9) | 4 | 2.5 (0.0, 5.7) |  |
| No adult caregiver | – | – | 1 | 0.6 (0.0, 1.9) |  |
| N/A | 14 | 5.7 | – | – |  |
| Number of children in household - mean | 2.7 (2.4, 3.1) | | 3.3 (2.7, 3.8) | | <0.09 |
| Help care for children in household | 218 | 89.3 (82.1, 96.6) | 144 | 88.9 (81.2, 96.6) | 0.317 |
| Parental/caregiver connectedness^a^ |  |  |  |  |  |
| Feel that parents care | 207 | 85.2 (74.8, 95.6) | 156 | 96.3 (92.4, 100.0) | **<0.001** |
| Feel that parents listen | 211 | 86.8 (79.3, 94.4) | 145 | 89.5 (82.5, 96.5) | 0.573 |
| Parental/caregiver monitoring^b^ |  |  |  |  |  |
| Parents/caregiver know friends | 129 | 53.1 (38.5, 67.6) | 76 | 47.2 (26.4, 68.0) | 0.602 |
| Parents/caregiver monitor time | 179 | 73.4 (62.2, 85.7) | 90 | 55.9 (42.4, 69.4) | **<0.028** |
| ≥1 close friends | 240 | 98.4 (96.7, 100.0) | 160 | 98.8 (97.1, 100.0) | 0.735 |
| ≥1 close opposite sex friend |  |  |  |  |  |
| Boys (female friend) | 43 | 36.4 (24.9, 48.0) | 36 | 48.6 (25.4, 71.9) | 0.289 |
| Girls (male friend) | 41 | 32.5 (19.6, 45.5) | 41 | 46.6 (27.8, 65.4) | 0.166 |
| Perceived peer norms about boy/girl relationships |  |  |  |  |  |
| Ok talk/spend time | 90 | 36.9 (19.2, 54.5) | 73 | 45.1 (21.4, 68.7) | 0.544 |
| Ok spend time alone | 57 | 23.4 (8.2, 38.5) | 63 | 38.9 (15.2, 62.6) | 0.221 |
| Ok physical contact | 24 | 9.8 (3.5, 16.1) | 43 | 26.5 (9.9, 47.4) | **0.052** |
| **School** |  |  |  |  |  |
| Ever attended school | 233 | 95.5 (91.7, 99.3) | 152 | 93.8 (88.8, 98.8) | 0.562 |
| Currently enrolled in school | 207 | 88.8 (83.0, 94.7) | 145 | 95.4 (91.7, 99.1) | 0.089 |
| Days of school missed past week^c^ |  |  |  |  |  |
| None | 119 | 57.5 (40.3, 74.6) | 82 | 56.5 (40.2, 72.9) | 0.841 |
| 1-2 | 59 | 28.5 (14.8, 42.2) | 47 | 32.4 (15.2, 49.6) |  |
| 3 or more | 29 | 14.0 (6.2, 22.0) | 16 | 11.0 (0.3, 21.7) |  |

* Pearson’s Chi Square statistic was used to test for the difference in proportions between those with vs. without someone else present during the interview.

^a)^ Proportion agreeing with the statements “I feel that my parents/guardians care about me”(feel that parent/guardian care) and “I feel like I can share personal things with one of my parents/guardians and she or he will listen” (feel that parent/guardian listen).

^b)^ Proportion agreeing that their parents (to some extent or completely) know who their friends are (parents monitor friends), and what they do with their free time (parents monitor time).

^c)^ Days of school missed during past week were only asked of those currently enrolled

**Additional file, Table 2.** Pubertal transitions and access to SRH information among Somali VYA: Sensitivity analysis by whether someone else was present during the interview

|  | **Boys** | | | |  | **Girls** | | | |  |
| --- | --- | --- | --- | --- | --- | --- | --- | --- | --- | --- |
|  | No one else present | | Someone else present | |  | No one else present | | Someone else present | |  |
|  | n | % (95 % CI) | n | % (95 % CI) | *p** | n | % (95 % CI) | n | % (95 % CI) | *p** |
| **Pubertal status, by age** |  |  |  |  |  |  |  |  |  |  |
| ***10-12 years*** |  |  |  |  |  |  |  |  |  |  |
| Did not start puberty | 40 | 76.9 (54.6, 99.2) | 29 | 72.5 (49.2, 96.0) | 0.649 | 46 | 74.2 (57.2, 91.5) | 36 | 66.7 (36.2, 97.1) | 0.537 |
| Started puberty (boys) | – | – | 1 | 2.5 (0.0, 8.1) |  |  |  |  |  |  |
| Started puberty (girls) |  |  |  |  |  | – |  | 2 | 3.7 (0.0, 9.6) |  |
| Do not know | 12 | 23.1 (0.8, 45.4) | 10 | 25 (1.8, 48.2) |  | 16 | 25.8 (8.8, 42.8) | 16 | 29.6 (0.0, 61.2) |  |
| ***13-14 years*** |  |  |  |  |  |  |  |  |  |  |
| Did not start puberty | 44 | 66.7 (42.5, 90.8) | 23 | 67.7 (40.8, 94.6) | 0.591 | 48 | 75.0 (49.3, 100.0) | 16 | 47.1 (7.7, 86.4) | 0.073 |
| Started puberty (boys) | 12 | 18.2 (0.0, 36.7) | 3 | 8.8 (0.0, 18.8) |  |  |  |  |  |  |
| Started puberty (girls) |  |  |  |  |  | 8 | 12.5 (0.9, 24.0) | 16 | 47.1 (5.2, 88.9) |  |
| Do not know | 10 | 15.1 (0.0, 32.2) | 8 | 23.5 (0.0, 50.4) |  | 8 | 12.5 (0.0, 28.4) | 2 | 5.8 (0.0, 15.6) |  |
| **Pubertal change reactions^a^** |  |  |  |  |  |  |  |  |  |  |
| Like becoming a woman/man |  |  |  |  |  |  |  |  |  |  |
| Disagree | 2 | 16.7 (0.0, 36.9) | – | – |  | – | ­– | – | – |  |
| Agree | 10 | 83.3 (63.0, 100.0) | 4 | 100.0 (–) | 0.202 | 8 | 100.0 (–) | 18 | 100.0 (–) | – |
| Feel comfortable with body changes |  |  |  |  |  |  |  |  |  |  |
| Disagree | 3 | 25.0 (4.5, 45.5) | – | – | 0.169 | – | – | 1 | 5.6 (0.0, 23.5) | 0.646 |
| Agree | 9 | 75.0 (54.5, 95.5) | 4 | 100.0 (–) |  | 8 | 100.0 (–) | 17 | 94.4 (76.5, 100.0) |  |
| Treated differently by parents due to body changes |  |  |  |  |  |  |  |  |  |  |
| Disagree | 9 | 75.0 (54.5, 95.5) | 1 | 25.0 (0.0, 82.2) | ***0.093*** | 4 | 50.0 (0.0, 100.0) | 1 | 5.6 (0.0, 23.5) | ***0.057*** |
| Agree | 3 | 25.0 (4.5, 45.5) | 3 | 75.0 (17.8, 100.0) |  | 4 | 50.0 (0.0, 100.0) | 17 | 94.4 (76.5, 100.0) |  |
| **Menstrual hygiene^a^** |  |  |  |  |  |  |  |  |  |  |
| Sufficient water and soap |  |  |  |  |  | 4 | 50.0 (2.7, 97.3) | 6 | 33.3 (10.0, 56.6) | 0.482 |
| No |  |  |  |  |  | 4 | 50.0 (2.7 (97.3) | 12 | 66.7 (43.3, 90.0) |  |
| Yes |  |  |  |  |  |  |  |  |  |  |
| Private washing facilities |  |  |  |  |  |  |  |  |  |  |
| No |  |  |  |  |  | 8 | 100 (–) | 13 | 72.2 (46.0, 98.4) | 0.130 |
| Yes |  |  |  |  |  | – | – | 5 | 27.8 (1.6, 54.0) |  |
| Cloth/pads |  |  |  |  |  |  |  |  |  |  |
| No |  |  |  |  |  | 6 | 75.0 (36.4, 100.0) | 15 | 83.3 (63.8, 100.0) | 0.646 |
| Yes |  |  |  |  |  | 2 | 25.0 (0.0, 63.6) | 3 | 16.7 (0.0, 36.2) |  |
| All hygiene items |  |  |  |  |  |  |  |  |  |  |
| No |  |  |  |  |  | 8 | 100.0 (–) | 18 | 100.0 (–) | **–** |
| Yes |  |  |  |  |  | – | – | – | – |  |
| **SRH information** |  |  |  |  |  |  |  |  |  |  |
| Learnt about body changes before these occurred^a^ |  |  |  |  |  |  |  |  |  |  |
| No | 3 | 25.0 (4.5, 45.5) | 2 | 50.0 (0.0, 100.0) | 0.366 | 1 | 12.5 (0.0, 31.8) | – | – | ***0.056*** |
| Yes | 9 | 75.0 (54.5, 95.6) | 2 | 50 (0.0, 100.0) |  | 7 | 87.5 (68.2, 100.0) | 18 | 100.0 (–) |  |
| Do not know |  |  |  |  |  |  |  |  |  |  |
| Access to information needed to understand body changes |  |  |  |  |  |  |  |  |  |  |
| Disagree | 22 | 18.6 (7.1, 30.2) | 28 | 37.8 (15.1, 60.6) | 0.114 | 25 | 19.8 (6.7, 33.0) | 37 | 42 (14.5, 69.6) | 0.113 |
| Agree | 92 | 78 (66.8, 89.1) | 44 | 59.5 (36.2, 82.7) |  | 96 | 76.2 (62.1, 90.3) | 47 | 53.4 (25.6, 81.2) |  |
| N/A | 4 | 3.4 (0.0, 7.5) | 2 | 2.7 (0.0, 6.6) |  | 5 | 4 (0.0, 8.8) | 4 | 4.6 (0.0, 10.8) |  |
| Wish had access to more information about body changes |  |  |  |  |  |  |  |  |  |  |
| Disagree | 16 | 13.6 (2.9, 24.2) | 10 | 13.5 (2.3, 24.8) | 0.887 | 12 | 10.3 (0.5, 20.1) | 9 | 10.2 (0.0, 24.1) | 0.423 |
| Agree | 97 | 82.2 (71.3, 93.1) | 62 | 83.8 (21.7, 95.8) |  | 108 | 85.7 (76.1, 95.3) | 79 | 89.8 (75.9, 100.0) |  |
| N/A | 5 | 4.2 (0.7, 7.8) | 2 | 2.7 (0.0, 8.6) |  | 5 | 4.0 (0.3, 7.6) | – | – |  |
| Learnt about pregnancy |  |  |  |  |  |  |  |  |  |  |
| No | 99 | 83.9 (72.7, 95.1) | 48 | 64.9 (41.2, 88.4) | ***0.094*** | 113 | 89.7 (83.1, 96.2) | 60 | 68.2 (43.2, 93.1) | **0.016** |
| Yes | 17 | 14.4 (3.7, 25.1) | 25 | 33.8 (10.0, 57.6) |  | 11 | 8.7 (2.3, 15.1) | 28 | 31.8 (6.9, 56.8) |  |
| Do not know | 2 | 1.7 (0.0, 4.2) | 1 | 1.3 (0.0, 4.3) |  | 2 | 1.6 (0.0, 3.8) | – | – |  |

*Pearson’s Chi Square statistic was used to test for the difference in proportions between those with vs. without someone else present during the interview.

^a)^ Among those who reported starting puberty.

**Additional file, Table 3a.** Information sources about SRH and general health among Somali VYAs: Sensitivity analysis by whether someone else was present during the interview (Girls)

|  | **Girls (n=214)** | | | | | | | |
| --- | --- | --- | --- | --- | --- | --- | --- | --- |
|  | **Main puberty info source** | | **Want more puberty info from** | | **Pregnancy info source^a,b^** | | **Seek health advice from^b^** | |
|  | No one else present | Someone else present | No one else present | Someone else present | No one else present | Someone else present | No one else present | Someone else present |
|  | % (95% CI) | % (95% CI) | % (95% CI) | % (95% CI) | % (95% CI) | % (95% CI) | % (95% CI) | % (95% CI) |
| Mother | **80.2**  (69.5, 90.9) | **67.1**  (50.4, 83.7) | **20.6**  (10.5, 30.8) | **17.1**  (0.0, 34.1) | 100.0 (–) | **89.3**  (60.9, 100.0) | **85.7**  (76.2, 95.2) | **79.5**  (62.8, 96.3) |
| Father | **5.6**  (0.0, 11.4) | **14.8**  (2.2, 27.3) | 3.2  (0.3, 6.0) | 3.4  (0.0, 7.2) | **45.4**  (15.2, 75.7) | 28.6  (2.3, 54.8) | **61.1**  (47.2, 74.9) | **64.8**  (52.2, 77.3) |
| Brother/sister | 4.0  (0.0, 8.8) | 5.7  (0.0, 11.2) | **19.1**  (11.2, 26.9) | **20.5**  (5.8, 35.1) | **45.4**  (15.2, 75.7) | **35.7**  (11.5, 59.9) | **61.1**  (42.2, 80.0) | **56.8**  (40.9, 72.8) |
| Other relative | – | 1.1  (0.0, 3.7) | 2.4  (0.0, 5.2) | 4.6  (0.0, 9.6) | 18.2  (0.0, 40.6) | 10.7  (0.0, 31.2) | 4.7  (0.0, 10.0) | 11.4  (0.0, 25.4) |
| Teacher | 0.8  (0.0, 2.8) | – | 11.1  (5.5, 16.8) | 2.3  (0.0, 7.0) | 18.2  (0.0, 43.7) | 10.7  (0.0, 32.9) | 10.3  (5.1, 15.6) | 6.8  (0.0, 13.5) |
| Friend(s) | **5.6**  (0.2, 10.9) | 6**.8**  (0.0, 15.0) | **30.9**  (16.0, 45.9) | **34.1**  (18.0, 50.2) | 27.3  (0.0, 58.4) | **50.0**  (4.0, 95.0) | 33.3  (22.2, 44.4) | 47.8  (32.9, 62.6) |
| Religious leader | – | – | 4.8  (0.3, 9.3) | 4.6  (0.0, 10.9) | – | 3.6  (0.0, 13.0) | 8.7  (1.8, 15.7) | 3.4  (0.0, 7.2) |
| Doctor/nurse | – | – | 0.8  (0.0, 2.5) | 10.2  (3.3, 23.8)* | – | – | 15.9  (3.0, 28.8) | 21.6  (0.0, 44.2) |
| Media^c^ | – | 2.3  (0.0, 7.5) | – | – |  |  |  |  |
| Other | – | – | – | – | – | – | – | 1.1  (0.0, 3.7) |
| Do not know | – | – | 2.4  (0.0, 4.9) | – | – | – | – | – |
| Did not learn | 4.0  (0.0, 10.6) | 2.3  (0.0, 7.5) | 4.8 (0.0, 11.5) | 3.4 (0.0, 9.3) |  |  |  |  |
| No one |  |  |  |  |  |  | – | – |

±p<0.1, * p<0.05, ** p<0.01, ***p<0.001 using Pearson’s Chi Square statistic to test for the difference in proportions between those with vs. without someone else present during the interview.

^a)^ Asked of those who reported learning about pregnancy (n=39 girls).

^b)^ Respondents could select more than one option (total exceeds 100%).

^c)^ Media includes books/magazines, films/video and Internet.

**Additional file, Table 3b.** Information sources about SRH and general health among Somali VYAs: Sensitivity analysis by whether someone else was present during the interview (Boys)

|  | **Boys (n=192)** | | | | | | | |
| --- | --- | --- | --- | --- | --- | --- | --- | --- |
|  | **Main puberty info source** | | **Want more puberty info from** | | **Pregnancy info source^a,b^** | | **Seek health advice from^b^** | |
|  | No one else present | Someone else present | No one else present | Someone else present | No one else present | Someone else present | No one else present | Someone else present |
|  | % (95% CI) | % (95% CI) | % (95% CI) | % (95% CI) | % (95% CI) | % (95% CI) | % (95% CI) | % (95% CI) |
| Mother | **61.8**  (44.9, 77.2) | **60.8**  (40.3, 81.3) | 9.3  (0.7, 17.9) | **13.5**  (0.0, 29.8) | **70.6**  (50.3, 90.9) | **68.0**  (30.5, 100.0) | **82.2**  (74.5, 90.0) | **75.7**  (61.1, 90.2) |
| Father | **14.4**  (6.4, 22.4) | **21.6**  (0.5, 42.8) | **14.4**  (5.5, 23.3) | 4.1  (0.0, 8.7)* | **70.6**  (51.4, 89.7) | **40.0**  (16.8, 61.2) | **69.5**  (59.5, 79.4) | **66.2**  (51.2, 81.2) |
| Brother/sister | **10.2**  (5.0, 15.3) | **6.8**  (0.0, 14.3) | **30.5**  (59.3, 79.6) | **20.3**  (6.2, 34.3) | **58.8**  (36.8, 80.9) | 40.0  (22.5, 57.5) | **63.6**  (51.1, 75.0) | **39.2**  (26.8, 51.6)* |
| Other relative | 2.5  (0.0, 6.1) | – | 4.2  (0.0, 9.8) | 9.5  (0.0, 20.4) | 11.8  (0.0, 28.9) | 12.0  (0.0, 25.8) | 15.2  (5.5, 25.0) | 21.6  (8.2, 35.0) |
| Teacher | – | – | 4.2  (0.0, 8.7) | 8.1  (0.7, 15.5) | 41.2  (19.1, 63.2) | 24.0  (0.0, 56.6) | 8.5  (2.8, 14.2) | 18.9  (5.4, 32.3) |
| Friend(s) | 8.5  (0.0, 19.1) | 5.4  (0.0, 11.7) | **27.1**  (12.6, 41.7) | **21.6**  (10.7, 32.5) | 35.3  (13.1, 57.5) | **64.0**  (28.3,99.7) | 32.2  (23.1, 41.3) | 37.8  (19.8, 55.9) |
| Religious leader | 0.8  (0.0, 2.5) | – | 3.4  (0.4, 6.5) | 1.3  (0.0, 4.2) | 5.6  (0.0, 19.3) | 4.0  (0.0, 13.6) | 8.5  (2.4, 14.5) | 6.7  (0.0, 13.5) |
| Doctor/nurse | – | – | 0.9  (0.0, 2.6) | 10.8  (0.0, 24.8)* | 5.6  (0.0, 16.5) | – | 9.3  (3.5, 15.1) | 16.2  (0.0, 37.9) |
| Media^c^ | – | 1.3  (0.0, 4.3) | 0.8  (0.0, 2.6) | – |  |  |  |  |
| Other | 0.8  (0.0, 2.6) | – | 0.9  (0.8, 2.5) | – | – | – | 2.5  (0.0, 6.2) | 1.3  (0.0, 4.1) |
| Do not know | 1.7  (0.0, 4.2) | 1.4  (0.0, 4.3) | 2.5  (0.0, 5.6) | 4.1  (0.0, 9.2) | – | – | – | – |
| Did not learn | – | 2.7  (0.0, 8.6) | 1.7  (0.0, 4.2) | 6.8  (0.0, 15.2) |  |  |  |  |
| No one |  |  |  |  |  |  | 0.8  (0.0, 2.6) | 1.3  (0.0, 4.1) |

±p<0.1, * p<0.05, ** p<0.01, ***p<0.001 using Pearson’s Chi Square statistic to test for the difference in proportions between those with vs. without someone else present during the interview.

^a)^ Asked of those who reported learning about pregnancy (n=42 boys).

^b)^ Respondents could select more than one option (total exceeds 100%).

^c)^ Media includes books/magazines, films/video and Internet.
